# One Simple Question Predicts Well‐Being in Radiation Therapy: The Role of Desire for Psychological Intervention and Perceived Social Support

**DOI:** 10.1002/jclp.23814

**Published:** 2025-06-04

**Authors:** Roni Laslo‐Roth, Sivan George‐Levi, Yael Galin Loncich, Myriam Sultan, Nirit Wiskop‐Farkash, Eli Sapir

**Affiliations:** ^1^ School of Behavioral Sciences Peres Academic Center Rehovot Israel; ^2^ Department of Psychology Achva Academic College Havat Shikmim Israel; ^3^ Radiation Oncology Department Samson Assuta Ashdod University Hospital Ashdod Israel

**Keywords:** anxiety, depression, life satisfaction, psycho‐oncological intervention, radiation therapy, social support

## Abstract

Radiation therapy (RT) is a critical part of cancer treatment often accompanied by heightened anxiety, depression, and reduced life satisfaction. Although psycho‐oncological (PO) interventions and social support can alleviate distress, it is unclear whether patients' desire for PO intervention reflects temporary or ongoing distress and whether all patients benefit from social support or only those seeking help. In this study we examined whether the desire for PO intervention during the start of RT predicted anxiety, depression, and life satisfaction at the start and end of RT and whether perceived social support moderated these effects. One‐hundred‐and‐forty‐six patients completed questionnaires at three time‐points (pre‐treatment, first treatment, and last treatment). Results showed that patients desiring PO intervention reported higher anxiety and depression at the start and end of RT. Higher perceived social support predicted lower depression and improved life satisfaction but only for patients expressing a desire for PO intervention. Screening for psychological distress is standard in oncology but often limited by time and resources during RT. The current study findings suggest that a single, straightforward question at the start of RT could identify at‐risk patients, enabling proactive intervention. Moreover, fostering perceptions of social support may help mitigate negative outcomes in the absence of professional intervention.

## 1 Introduction

Many patients experience psychological distress related to both their cancer diagnosis and the treatment process (Curran et al. 2017; Wang et al. 2020). During cancer treatment, up to two‐thirds of patients will, at some point in their disease trajectory, receive radiation therapy (RT) as a critical part of their treatment plan. The goals of RT include curing the disease, prolonging life, or alleviating symptoms (Chandra et al. 2021). However, RT often has negative effects on cancer patients, leading to long‐term physical challenges such as pain and decreased functioning, as well as significant emotional distress (Dilalla et al. 2020). Specifically, RT is associated with increased anxiety, depression, and reduced life satisfaction (Mungase et al. 2021). This high level of distress warrants clinical attention, as up to 25% of patients interrupt their RT treatment due to emotional challenges (Clover et al. 2011).

Evidence suggests that psycho‐oncological (PO) interventions effectively reduce distress, yet only a small proportion of patients seek PO support. When they do, it is typically early in the RT process (Guo et al. 2013; Holland 2018; Sanjida et al. 2018). Given that distress and life satisfaction levels fluctuate significantly throughout RT (Hess and Chen 2014), it is unclear whether patients' initial desire for PO intervention predicts their distress levels over the course of treatment or merely reflects temporary distress experienced at the beginning of RT.

PO intervention is not the only effective form of support for patients undergoing RT. Patients' perceptions of social support from family, friends, and healthcare professionals (e.g., nurses, oncologists, and radiation therapists) play a vital role in helping them cope with cancer, reduce distress, and enhance overall well‐being (Cohen and McKay 2020; Fong et al. 2017; Riedl et al. 2018). However, the effectiveness of perceived social support depends on how well it aligns with the patient's actual needs and desire for assistance (Zee et al. 2020).

Therefore, we had two aims in the current study: (1) to explore whether patients' wish for PO intervention would be associated with greater distress (anxiety and depression symptoms) and lower levels of life satisfaction at the beginning of RT treatment and at the end of treatment, and (2) to examine whether perceived social support would be a more critical factor in predicting reduced distress and improved life satisfaction, at the end of RT, for patients who wished for PO intervention (had a greater desire for help) than for those who did not.

### 1.1 Anxiety, Depression, and Life Satisfaction During RT

It is well‐documented that 30%−49% of patients attending RT appointments experience anxiety and distress (Hess and Chen 2014; Shankar et al. 2016). They also often experience a decline in life satisfaction, a key indicator of well‐being and quality of life in cancer patients (Chambers et al. 2017; Kovyazina et al. 2021; Polański et al. 2020). Several studies have examined the trajectories of anxiety and depression in cancer patients undergoing RT. Research indicates that anxiety levels are typically highest before the initiation of RT and tend to decrease over the course of treatment (Burns et al. 2022). Conversely, depressive symptoms may persist throughout the treatment period and even months after treatment ends (Chen et al. 2020; Hess and Chen 2014). Although life satisfaction commonly declines due to the negative impacts of cancer, certain aspects of it may improve over time (Fernández‐Sánchez et al. 2024).

Emotional distress during RT can reduce treatment compliance, prolong hospitalization, and exacerbate the physical effects of cancer (Rangarajan and Jayaraman 2017). Contributing factors include perceived risks of radiation, the experience of lying by oneself under a large machine, the need to remain still during treatment, and concerns about side effects such as fatigue, skin changes, and nausea (Nayak et al. 2022).

Although emotional distress is common among cancer patients undergoing RT, it is not inevitable. PO interventions have proven effective in alleviating distress in these patients (Guo et al. 2013; Holland 2018; Sanjida et al. 2018). However, not all distressed patients seek PO intervention. There remains a need to clarify the relation between patients' desire for PO intervention and their distress and life satisfaction levels, particularly at different stages of RT (Parmet et al. 2023).

### 1.2 The Desire for PO Intervention

The percentage of cancer patients expressing a desire for PO intervention to address psychosocial concerns related to their diagnosis and treatment ranges from 13% to 60% across studies (Baker‐Glenn et al. 2011; Faller et al. 2016; Schulze et al. 2022). Specifically, studies conducted during RT have found that 13%−25% of patients wish to receive PO intervention (Adeberg et al. 2021; Riedl et al. 2018).

Most studies indicate that cancer patients who wish for PO intervention report higher levels of emotional distress (e.g., Admiraal et al. 2020; Baker‐Glenn et al. 2011; Bergerot et al. 2021). For instance, a study involving 1340 cancer patients found that 66% did not seek PO support referral, but those who did were significantly more distressed (Admiraal et al. 2020). Similarly, in another study a modest association was observed between the desire for PO intervention and the severity of anxiety and depression in 128 cancer patients (Baker‐Glenn et al. 2011). Bergerot et al. (2021) also identified a significant link between requests for PO assistance and reported distress. However, in some studies no relation was found between patients’ distress levels and their wish for PO intervention (Brebach et al. 2016; Clover et al. 2011; Merckaert et al. 2010; Tondorf et al. 2018).

In the specific context of RT, the connection between the desire for PO intervention and patients' reported distress and life satisfaction has been less thoroughly explored (Riedl et al. 2018). As distress and life satisfaction levels can fluctuate throughout RT (Hess and Chen 2014), it is important to determine whether the desire for PO intervention consistently correlates with distress and life satisfaction at different treatment stages. Additionally, it is crucial to assess whether an initial desire for PO intervention predicts distress and life satisfaction throughout RT or merely reflects the initial distress and reduced life satisfaction commonly observed early in treatment.

If patients desiring PO intervention indeed experience greater distress and lower life satisfaction over time, further investigation into additional support resources is essential. Perceived support from family, friends, and healthcare professionals (e.g., oncologists, nurses, and radiation therapists) can play a significant role in reducing distress and improving well‐being (Riedl et al. 2018). Such perceived support is especially critical during RT, as relationships with family and friends may face strain during cancer treatment and recovery (Fong et al. 2017). Additionally, RT patients often report deficits in perceived social support, underscoring the need for comprehensive psychosocial interventions (Riedl et al. 2018).

### 1.3 Perceived Social Support

Perceived social support is defined as the subjective judgment of being loved, valued, and connected within a network, as well as having access to emotional and practical assistance from family, friends, and community (Wills 1998). The concept emphasizes the belief that confidence in close and meaningful interpersonal relationships serves as a critical resource for emotional well‐being (Taylor 2011). Evidence suggests that the perception of available social support is a stronger predictor of positive health outcomes than the actual receipt of support, as explicit support may sometimes undermine recipients' coping efforts (Haber et al. 2007).

Perceived social support is regarded as a vital coping resource during periods of illness, functioning as a stress‐buffering mechanism (Cohen and McKay 2020). Such support has been shown to enhance life satisfaction and reduce anxiety and depression among cancer patients (Gonzalez‐Saenz de tejada et al. 2017; Sayılan and Doğan 2020; Zamanian et al. 2021). Additionally, perceived social support has been reported to have a protective effect against the development of distress during cancer treatment (Usta 2012).

### 1.4 The Current Study

In the present study we had two main aims. First, we sought to explore whether patients' initial wish for PO intervention at the beginning of RT treatment would predict their distress and life satisfaction levels at the time of the request for PO intervention, as well as over time, specifically at the end of RT treatment. The second aim was to examine the interplay between RT patients’ initial wish for PO intervention and their perceived social support levels in predicting distress and life satisfaction at the end of RT.

RT patients perceive support from various sources, including close family members, peers, and specialist staff who provide care during radiation treatment. These staff members include radiation oncologists, radiation therapists, nurses, medical physicists, and other healthcare professionals (Arnold et al. 2021). Research suggests that social support is most effective when it aligns with the specific needs of recipients, such as their desire for assistance (Zee et al. 2020).

An additional objective of the current study was to investigate whether perceived social support would be more beneficial in predicting reduced distress and increased life satisfaction among patients who expressed a wish for PO intervention (indicating a greater need for help and support) than among those who did not express such a wish.

## 2 Methods

### 2.1 Study Design and Population

The current study was part of a large‐scale longitudinal and prospective investigation into psychological coping among patients undergoing RT. The target population consisted of cancer outpatients treated at the Department of Radiology at Assuta Ashdod Hospital in Israel.

Eligible patients were recruited between January 2022 and September 2023 from 1,862 patients treated daily in the radiation department over a treatment period of approximately three to 6 weeks. Exclusion criteria included patients whose mother tongue was not Hebrew, those with a total treatment duration of less than 2 weeks (at least 10 treatment fractions, i.e., radiation doses), and those who couldn't fill out the questionnaires due to their physical functioning.

Of the 223 eligible patients who were invited to take part in the study, 66 declined (29.6%), resulting in a final sample of 146 patients (58.7% recruitment rate). Approval for the study was obtained from the hospital's review board. Radiation therapists approached all eligible patients, explained the study's purpose, and invited them to volunteer. Informed consent was obtained from participants before their inclusion.

### 2.2 Study Procedure

Participants completed self‐report questionnaires at three time‐points:

- Time 1 (Pre‐Treatment Preparation): On the day of the simulation (pre‐treatment preparation), participants completed the Hospital Anxiety and Depression Scale (HADS) and a life satisfaction scale. They were also asked, “Would you like to schedule a session with a psychologist?” (yes/no). Patients' PO intervention requests were shared with the PO staff in the RT department.
- Time 2 (First Radiation Fraction): Participants completed a social support scale.
- Time 3 (End of RT Course): At the conclusion of RT (approximately three to 6 weeks after the first treatment), participants once again completed the HADS and life satisfaction scales.

### 2.3 Participant Retention

Of the initial 146 participants, 32 discontinued their participation at Time 2 or Time 3, resulting in an attrition rate of 21.9%. Ultimately, 114 participants completed all three rounds of questionnaires.

### 2.4 Instruments

#### 2.4.1 HADS

The HADS was used to assess depressive and anxious symptomatology among the study participants. This instrument has been validated in multiple studies, including those conducted with hospitalized patients in secondary care settings and with cancer patients (Naser et al. 2021). The HADS consists of 14 items, each rated on a 4‐point Likert scale, where responses range from 0 (*not at all*) to 3 (*most of the time*), depending on the specific item.

Higher scores indicate greater severity of symptoms on the respective subscales. The Cronbach's *α* coefficients for the depression subscale were 0.77 at Time 1 and 0.80 at Time 3. For the anxiety subscale, the Cronbach's *α* coefficients were 0.82 at Time 1 and 0.88 at Time 3.

#### 2.4.2 Satisfaction with Life Scale (SWLS)

Life satisfaction was measured using the SWLS, developed by Diener and colleagues (Diener et al. 1985). This scale includes five items rated on a 7‐point Likert scale, ranging from 1 (*strongly disagree*) to 7 (*strongly agree*). The total life satisfaction score was calculated by averaging the responses to the five items. The Cronbach's *α* reliability coefficient for the SWLS was 0.85 at Time 1 and 0.87 at Time 3.

### 2.5 Multidimensional Scale of Perceived Social Support (MSPSS)

Perceived social support was measured via use of the MSPSS (Zimet et al. 1988). The scale includes 12 items describing perceived support from family, friends, and significant others (e.g., “I can talk about my problems with my family”; “My friends really try to help me”). Items are ranked on a 7‐point Likert scale, ranging from 1 (*strongly disagree*) to 7 (*strongly agree*). In the current study, three four‐item subscales were used: family support (e.g., “I can talk about my problems with my family.”), friend support (e.g., “I can rely on my friends when I have problems.”), and an adaptation of the subscale of support from significant others to support from the radiotherapy staff (“There is someone in the radiotherapy department who helps me when I need it”). The Cronbach's *α* coefficients for the total perceived social support was 0.84.

### 2.6 Demographic and Clinical Data

Demographic data included participants’ age, gender, years of education, and family status. Clinical data, obtained from medical records, included the following:

- Tumor type and stage
- Surgical removal of tumor (yes/no)
- Chemotherapy (yes/no)
- Karnofsky Performance Scale (KPS): A measure of functional impairment ranging from 0 to 100, with lower scores indicating worse survival outcomes for severe illnesses
- Number of radiation sessions
- History of cancer in the family
- Presence of comorbidities

### 2.7 Data Analysis

First, baseline demographic and clinical characteristics of the two groups of patients—those who wanted PO intervention (PO group) and those who did not want PO intervention (non‐PO group)—were analyzed.

Second, to examine differences between these two groups regarding the research variables while controlling for a Type I error, two multivariate analyses of variance (MANOVAs) were conducted with patient group (PO vs. non‐PO) serving as the independent variable:

1. In the first MANOVA, the dependent variables were patients' anxiety, depression, and life satisfaction at Time 1 (T1) and perceived support at Time 2 (T2).
2. In the second MANOVA, the dependent variables were patients' anxiety, depression, and life satisfaction at Time 3 (T3).

Next, Pearson correlations were performed to examine the associations between continuous demographic variables (patients’ age, years of education, cancer stage, and number of RT treatments) and the research variables (anxiety, depression, and life satisfaction at T1 and T3, as well as perceived support).

Subsequently, three multiple hierarchical regression analyses were conducted to investigate whether the PO group, social support, and their interaction predicted patients' anxiety, depression, and life satisfaction at T3, beyond their corresponding levels at T1. For these analyses:

1. The first regression used patients' anxiety at T3 as the criterion variable.
2. The second regression used patients' depression at T3 as the criterion variable.
3. The third regression used patients' life satisfaction at T3 as the criterion variable.

Finally, to examine the moderating role of wish for PO intervention group in the relation between social support and patients' anxiety, depression, and life satisfaction at T3 (controlling for levels at T1), three moderation models were tested using Model 1 in PROCESS v3.5 (Hayes 2018). This approach identified both direct and indirect effects. The moderation analyses employed the bootstrapping method with bias‐corrected confidence estimates (Preacher and Hayes 2008). In this study, a 95% confidence interval for the indirect effects was generated using 5000 bootstrap resamples.

## 3 Results

### 3.1 Characteristics of the Sample

Baseline characteristics of the two groups of patients, PO (50% of the participants) and non‐PO, are presented in Table 1. Only one participant received psychological treatment at the end of the RT. In the PO group, patients' ages ranged from 36 to 77 years, their average age was 56.17 years (SD = 8.15), their average years of education was 14.73 years (SD = 3.47), and their average number of radiation treatment fractions was 13.67 (SD = 5.29). In the non‐PO group, ages ranged from 34 to 75 years, their average age was 52.72 years (SD = 8.49), their average years of education was 14.58 years (SD = 3.39), and their average number of radiation treatment fractions was 13.75 (SD = 4.99). The majority of patients in both groups were women, single or married. As for clinical characteristics, the prevalent type of cancer in both groups was breast cancer and the most prevalent cancer stage was 1 (60.4%). For most patients the KPS score was high (95 Karnofsky index), and most patients had a history of cancer in the family. Most patients were not treated with chemotherapy (63.1%). Of the patients, 46.4% had comorbidity, most with hypertension and diabetes.

**Table 1:** Sociodemographic and clinical characteristics of the sample.

Characteristics	PO group *M* (SD)	Non‐PO group *M* (SD)	*p*
Age	55.39 (11.30)	56.00 (11.90)	0.765
Years of education	14.73 (3.74)	14.58 (3.39)	0.805
Number of radiation treatments	13.67 (5.29)	13.75 (4.99)	0.938

Characteristics	PO group *n* (%)	Non‐PO group *n* (%)	*p*
*Gender*			0.142
Male	3 (4.5%)	8 (12.1%)	
Female	59 (89.4%)	58 (87.9%)	
*Family status*			0.249
Single	25 (37.9%)	22 (33.3%)	
Married	24 (36.4%)	34 (51.5%)	
Divorced/separated	4 (6.1%)	6 (9.1%)	
Widowed	6 (9.1%)	2 (3%)	
*Tumor type*			0.315
Breast cancer	57 (86.4%)	57 (86.4%)	
Prostate cancer	1 (1.5%)	4 (6.1%)	
Lung cancer	1 (1.5%)	—	
Head and neck cancer	2 (3%)	1 (1.5%)	
Uterine cancer	—	1 (1.5%)	
Lymphoma	—	2 (3%)	
Colon cancer	1 (1.5%)	—	
*Surgery before radiation therapy*			0.104
Yes	26 (39.4%)	36 (54.5%)	
No	34 (51.1%)	26 (39.4%)	
*Cancer stage*			0.112
0	6 (9.2%)	2 (3.3%)	
1	42 (64.6%)	35 (57.4%)	
2	10 (15.4%)	14 (23%)	
3	5 (7.7%)	8 (13.%)	
4	3 (3.1%)	3 (3.3%)	
*Karnofsky index*			0.697
75	3 (4.5%)	4 (6.1%)	
95	56 (84.8%)	55 (83.3%)	
*Chemotherapy*			0.182
Yes	25 (43.1%)	16 (30.8%)	
No	33 (56.9%)	36 (69.2%)	
*Comorbidities*			0.602
Yes	30 (45.5%)	30 (45.5%)	
No	36 (54.5%)	36 (54.5%)	
*Family history*			0.684
Yes	41 (62.1%)	45 (68.2%)	
No	18 (27.3%)	15 (22.7%)	
Don't know	1 (1.5%)	2 (3%)	

To assure that the sample characteristics were fairly similar between groups, several comparisons were carried out using chi‐square and independent sample *t* tests to compare the two groups (PO vs. non‐PO). The two groups were found to be similar in terms of age, gender, years of education, and family status. The two groups were also found to be similar in their clinical background: tumor type and stage, surgical removal, chemotherapy, KPS, number of radiation treatments, history of cancer in the family, and other comorbidities.

Clinical depressive and anxious symptomatology were defined as a total score of >9 on the depression subscale and >7 on the anxiety subscale, respectively (Annunziata et al. 2020). The prevalence of depressive symptomatology among all patients at T1 was 8.5% and at T3, 10.2%. Clinical depressive symptomatology was more prevalent in the PO group at T1 (13.6%) than in the non‐PO group (1.5%). Depressive symptomatology was also more prevalent in the PO group at T3 (PO group 16.2%, non‐PO group 0%). The prevalence of clinical anxious symptomatology among all patients at T1 was 42.3% and at T3, 34.1%. Anxious symptomatology was more prevalent in the PO group at T1 (50%) than in the non‐PO group (34.8%). Anxious symptomatology was more prevalent in the PO group at T3 than in the non‐PO group (PO group 37.8%; non‐PO group 27.9%).

#### 3.1.1 Preliminary Analyses

First, two MANOVAs were performed with group (PO group vs. non‐PO group) serving as the independent variable. In the first MANOVA, the dependent variables were variables that were measured at the beginning of the RT treatment (T1 and T2): anxiety, depression, and life satisfaction at T1 and perceived social support at T2. In the second MANOVA, the dependent variables were variables that were measured at the end of the RT treatment (T3): anxiety, depression, and life satisfaction at T3.

The first MANOVA yielded a main effect for group, *F*(4, 95) = 3.91, *p* = 0.006, partial *η*² = 0.141. Means, SDs, and F scores of the univariate analysis are presented in Table 2. As expected, patients who wanted PO intervention reported higher levels of anxiety and depression at T1 than did patients who did not want PO intervention. However, unexpectedly, no significant differences were found between the groups regarding life satisfaction at T1 or perceived social support.

**Table 2:** Means, standard deviations, and F scores for ANOVAs between PO and non‐PO group at the beginning of treatment.

Variable	PO	Non‐PO	F(1, 98)	Partial *ƞ*²
Anxiety T1				
*M*	1.07	0.85	4.60*	0.045
SD	0.52	0.49		
Depression T1				
*M*	0.77	0.44	31.22**	0.119
SD	0.52	0.35		
Satisfaction T1				
M	5.44	5.71	2.08	0.021
SD	0.97	0.89		
Social support				
*M*	5.72	6.06	3.50	0.034
SD	1.00	0.74		

* *p* < 0.05;

** *p* < 0.01.

The second MANOVA yielded a main effect for group, *F*(3, 76) = 5.08, *p* = 0.003, partial *η*² = 0.167. Means, SDs, and F scores of the univariate analysis are presented in Table 3. As expected, patients who wanted PO intervention reported higher levels of anxiety and depression at T3 than did patients who did not want PO intervention. However, unexpectedly, no significant differences were found between the groups regarding life satisfaction.

**Table 3:** Means, standard deviations, and *F* scores for ANOVAs between PO and non‐PO group at the end of treatment.

Variable	PO	Non‐PO	*F*(1, 78)	Partial *ƞ*²
Anxiety T3				
*M*	1.03	0.78	4.30*	0.052
SD	0.59	0.48		
Depression T3				
*M*	0.73	0.36	13.65**	0.149
SD	0.56	0.31		
Satisfaction T3				
M	5.74	5.86	0.35	0.004
SD	1.09	0.72		

* *p* < 0.05;

** *p* < 0.01.

Pearson correlations between the research measures were calculated, revealing that whereas anxiety and depression at both time‐points (T1 and T3) were positively correlated, life satisfaction at both time‐points was negatively correlated with anxiety and depression at both time‐points. Perceived social support was positively correlated with life satisfaction at T1 and T3 and negatively correlated with depression at T3, but not with depression at T1 and not with anxiety at either time‐point. As for the demographic variables, age was found to be positively correlated with depression at T1 and with number of RT treatments. Cancer stage was not related to any of the variables. The results of the descriptive statistics and correlation analysis among the research measures are presented in Table 4.

**Table 4:** Means, SDs, and Pearson correlations among the variables.

Variables	1	2	3	4	5	6	7	8	9	10
1. Anxiety T1	—	0.50**	−0.41**	0.77**	0.54**	−0.41**	−0.05	0.20*	−0.09	−0.07*
2. Depression T1		—	−0.36**	0.37**	0.70**	−0.41*	−0.12	0.24**	0.02	−0.02
3. Satisfaction T1			—	−0.25*	−0.34**	0.79**	0.37**	−0.10	−0.01	−0.09
4. Anxiety T3				—	0.65**	−0.41**	−0.13	0.11	0.07	0.01
5. Depression T3					—	−0.54**	−0.31**	0.15	−0.07	0.01
6. Satisfaction T3						—	0.39**	−0.12	0.02	−0.05
7. Social support							—	−0.10**	−0.09	−0.05
8. Age								—	0.04	−0.24**
9. Years of education									—	−0.05
10. Number of RT treatments										—
*M*	3.61	2.69	4.91	2.81	1.82	5.14	3.95	60.58	15.03	21.09
SD	1.17	1.15	1.28	1.12	1.08	1.28	0.66	10.79	3.40	10.68

* *p* < 0.05;

** *p* < 0.01.

#### 3.1.2 Predictors of Patients' Anxiety, Depression, and Life Satisfaction

To examine whether PO group and perceived social support predicted anxiety, depression, and life satisfaction at T3, three multiple hierarchical regressions were performed, one for each dependent variable. All continuous variables were standardized using Z transformation before analysis.

### 3.2 Predictors of Anxiety

To examine whether PO group and perceived social support predicted anxiety at T3 beyond anxiety levels at T1, multiple hierarchical regression was performed with anxiety levels at T3 serving as the criterion variable. Step 1 examined the direct effects of demographic and clinical variables (age, gender, and number of RT treatments) and anxiety levels at T1. Step 2 added PO group and perceived social support. Step 3 added interaction terms between PO group and perceived social support. The data are shown in Table 5.

**Table 5:** Hierarchical regression analysis for predicting *anxiety* at T3.

*B*	Step 1	Step 2	Step 3
Patient age	0.08	0.06	0.05
Patient gender	0.08	0.07	0.08
Number of RT treatments	0.06	0.06	0.04
Anxiety T1	0.76**	0.75**	0.73**
Social support		−0.09	−0.07
PO group		0.04	0.04
Social support* PO group			−0.15
*R*²	0.575	0.585	0.606
Model p	0.000	0.000	0.000

* *p* < 0.05;

** *p* < 0.01.

Step 1 showed a main effect for anxiety levels at T1. Step 2 also showed positive associations between anxiety levels at T1 and anxiety levels at T3. Step 3 did not yield significant results for the effect of the interaction between PO group and perceived social support on anxiety levels at T3, beyond anxiety levels at T1. Thus, the results didn't support the hypotheses concerning the moderating role of PO group in the association between perceived social support and anxiety levels at T3.

### 3.3 Predictors of Depression

To examine whether PO group and perceived social support predicted depression at T3 beyond depression levels at T1, multiple hierarchical regression was performed with depression levels at T3 serving as the criterion variable. Step 1 examined the direct effects of demographic variables (age, gender, and number of RT treatments) and depression levels at T1. Step 2 added PO group and perceived social support. Step 3 added interaction terms between PO group and perceived social support. The data are shown in Table 6.

**Table 6:** Hierarchical regression analysis for predicting depression at T3.

*B*	Step 1	Step 2	Step 3
Patient age	0.04	0.01	−0.01
Patient gender	0.07	0.06	0.06
Number of RT treatments	−0.06	−0.06	−0.07
Depression T1	0.74**	0.68**	0.65**
Social support		−0.21*	0.19*
PO group		0.10	0.11
Social support* PO group			−0.17*
*R*²	0.549	0.605	0.632
Model p	0.000	0.000	0.000

* *p* < 0.05;

** *p* < 0.01.

The Step 1 data showed a main effect for depression levels at T1. Step 2 showed negative associations between perceived social support and depression levels at T3, and positive associations between depression levels at T1 and depression levels at T3. Step 3 showed that the interaction between PO group and perceived social support had a significant effect on depression levels at T3, beyond depression levels at T1, thus supporting the hypotheses concerning the moderating role of PO group in the association between perceived social support and depression levels at T3.

### 3.4 Predictors of Life Satisfaction

To examine whether PO group and perceived social support predicted life satisfaction at T3 beyond life satisfaction levels at T1, multiple hierarchical regression was performed with life satisfaction levels at T3 serving as the criterion variable. Step 1 examined the direct effects of the demographic variables (age, gender, and number of RT treatments) and life satisfaction levels at T1. Step 2 added PO group and perceived social support. Step 3 added interaction terms between PO group and perceived social support. The data are shown in Table 7.

**Table 7:** Hierarchical regression analysis for predicting *life satisfaction* at T3.

*B*	Step 1	Step 2	Step 3
Patient age	−0.02	−0.01	.02
Patient gender	−0.01	−0.01	−0.03
Number of RT treatments	−0.02	−0.01	.02
Life satisfaction T1	0.78**	0.74**	0.78**
Social support		0.12	0.06
PO group		0.01	0.01
Social support* PO group			0.27**
*R*²	0.612	0.627	0.705
Model p	0.000	0.000	0.000

* *p* < 0.05;

** *p* < 0.01.

The Step 1 data showed a main effect for life satisfaction levels at T1. Step 2 showed positive associations between life satisfaction levels at T1 and life satisfaction levels at T3. Step 3 showed that the interaction between PO group and perceived social support had a significant effect on life satisfaction levels at T3, beyond life satisfaction levels at T1, thus supporting the hypotheses concerning the moderating role of PO group in the association between perceived social support and life satisfaction levels at T3.

#### 3.4.1 Testing the Moderation Models

To examine whether PO moderated the relation between social support and depression and between social support and life satisfaction, two moderation models were tested (one for each as the independent variable) using PROCESS 3.4 (Command Model 1) (Hayes 2018). Interaction terms were computed using mean centering. Anxiety was not tested as a dependent variable, as the results of the regression did not yield a significant interaction.

**Figure 1:**
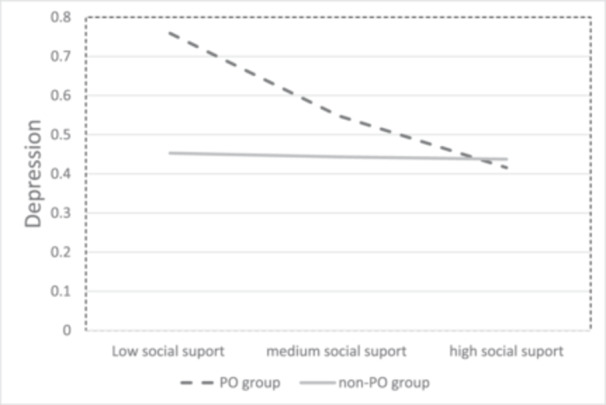
PO group moderates the relation between perceived social support and patients' depression.

In the first model, social support served as the independent variable, depression level at T3 as the dependent variable, and PO group as the moderating variable. Depression level at T1 was included as a covariate. The model indicated that social support was negatively related to depression levels at T3 such that high social support was linked to low levels of depression, *b* = −0.09, SE = 0.04, *t* = −2.08, *p* = 0.040, 95% CI = [−0.1768, −0.0034]. PO group was not significantly related to depression, *b* = 0.14, SE = 0.08, *t* = 1.76, *p* = 0.083, 95% CI = [−0.0185, 0.2914]. As expected, the results indicated that PO group significantly moderated the relation between social support and depression, *b* = −0.18, SE = 0.08, *t* = −2.09, *p* = 0.040, 95% CI = [−0.3503, 0.0080]. Analysis of the moderation effect indicated that whereas the relation between social support and depression levels at T3 (beyond depression levels at T1) was not significant for the non‐PO group, *b* = −0.01, SE = 0.06, *t* = −0.13, *p* = 0.985, 95%, CI = [−0.1353, 0.1186], the relation was significantly negative for the PO group, *b* = −0.19, SE = 0.06, *t* = −3.26, *p* = 0.002, 95% CI = [−0.3024, −0.0727]). In other words, social support was negatively related to depression levels at T3 only for those who wanted PO intervention but not for those who did not want such help (non‐PO group) beyond depression levels at T1, as depicted in Figure 1.

**Figure 2:**
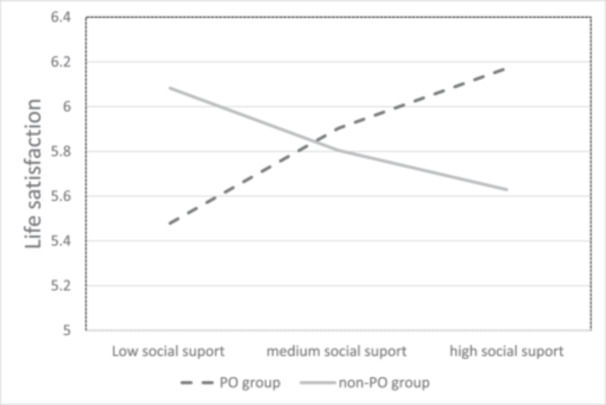
PO group moderates the relation between perceived social support and life satisfaction.

In the second model, social support served as the independent variable, life satisfaction level at T3 as the dependent variable, and PO group as the moderating variable. Life satisfaction level at T1 was included as a covariate. The model indicated that social support was not significantly related to life satisfaction levels at T3 (beyond life satisfaction levels at T1), *b* = −0.01, SE = 0.13, *t* = −0.08, *p* = 0.674, 95% CI = [−0.1406, 0.2159]. PO group was not significantly related to life satisfaction, *b* = 0.14, SE = 0.08, *t* = 1.76, *p* = 0.938, 95% CI = [−0.2814, 0.2602]. However, as expected, the results indicated that PO group significantly moderated the relation between social support and life satisfaction, *b* = 0.63, SE = 0.16, *t* = 3.96, *p* < 0.001, 95% CI = [0.3100, 0.9422]. Analysis of the moderation effect indicated that whereas the relation between social support and life satisfaction levels at T3 (beyond life satisfaction levels at T1) was not significant for the non‐PO group, *b* = −0.248, SE = 0.13, *t* = −1.93, *p* = 0.057, 95%, CI = [−0.5036, 0.0081], the relation was significantly positive for the PO group, *b* = 0.378, SE = 0.11, *t* = 3.53, *p* < 0.001, 95% CI = [0.1640, 0.5926]. In other words, social support was positively related to life satisfaction levels at T3 only for those who wanted PO intervention but not for those who did not want such help (non‐PO group), as depicted in Figure 2.

## 4 Discussion

The aim of the current study was to investigate whether patients who express a desire for PO intervention at the beginning of RT are more anxious, more depressed, and less satisfied both at the start and at the end of RT. Additionally, we examined whether perceived social support would predict reduced distress (anxiety and depression symptoms) and improved life satisfaction at the end of RT, particularly for patients who wanted PO intervention (vs. those who did not).

According to previous studies, cancer patients who express a desire for psychological intervention recognize their emotional distress (e.g., Admiraal et al. 2020; Baker‐Glenn et al. 2011; Bergerot et al. 2021). Likewise, in this study, patients who wanted PO intervention reported significantly higher levels of anxiety and depression symptoms than those who did not. However, there were no differences between the groups in terms of demographic or clinical variables, life satisfaction, or perceived social support, suggesting that the primary distinction was their level of distress.

The current study findings indicate that a desire for PO intervention at the start of RT reflects not only immediate distress but also persistent distress over time. Patients who wanted PO intervention experienced heightened anxiety and depression both at the beginning and at the end of RT, emphasizing the importance of early identification and intervention. These results suggest that distress in these patients is unlikely to resolve without clinical attention and ongoing support.

Previous studies conducted during RT reported that 13%–25% of patients expressed a desire for psychological intervention (Adeberg et al. 2021; Riedl et al. 2018), typically after initial distress screening. In contrast, in the current study patients were asked during the simulation phase, before treatment, whether they wanted psychological intervention. A notably higher percentage (50%) expressed a desire for intervention, likely influenced by the proactive opportunity to request assistance.

These results strongly suggest that a simple written question at the start of RT can serve as an effective measure of distress and may eliminate the need for more elaborate screening processes. This method has predictive value for identifying patients at risk for anxiety and depression. Furthermore, patients are often more receptive to interventions offered early and through less direct means, such as written or telephone‐based approaches, rather than face‐to‐face interactions (Brebach et al. 2016).

In this study, most participants were breast cancer patients undergoing a relatively short course of RT (approximately 3 weeks). Many did not receive PO intervention during treatment, highlighting the importance of identifying protective factors, such as perceived social support, that could mitigate distress.

Consistent with prior research (Gonzalez‐Saenz de tejada et al. 2017; Sayılan and Doğan 2020; Zamanian et al. 2021), in the current study perceived social support was negatively associated with depression and positively associated with life satisfaction. However, a key finding of this study was that the benefits of perceived social support were significant only for patients who expressed a desire for PO intervention. For these patients, higher levels of perceived social support were related to lower depression and improved life satisfaction at the end of RT. In contrast, perceived social support did not show similar benefits for patients who did not want PO intervention.

Several explanations may be suggested for the contribution of the interaction between perceived social support and the desire for PO intervention to patients' depression and satisfaction levels. First, this interaction may imply that patients who want PO intervention are generally more open to receiving help, proactive in managing their emotional well‐being, and likely to utilize social support effectively (Bickel et al. 2022; Coppini et al. 2023; Schuit et al. 2021). The second explanation aligns with the buffering hypothesis (Cohen and Wills 1985), suggesting that patients seeking psychological intervention experience heightened distress and thus benefit more from the buffering effects of social support. Another explanation relies on “optimal matching” (Cutrona 1990), which claims that support is most effective when it “matches” individuals' needs, such as their wish for help. Some studies even point to the mixed blessing of support, suggesting that despite the best intentions of family, friends, and health professionals, the availability of their support is not always helpful, and at times might even worsen patients' outcomes as such support may reduce patients' sense of competence and heighten dependency (Breuer et al. 2017; Sebri et al. 2021).

In the current study, perceived social support did not predict lower levels of anxiety symptoms over time. This finding aligns with previous research suggesting that anxiety during RT often stems from immediate concerns, such as treatment sessions and side effects (Andersen and Tewfik 1985; Gillan et al. 2014), which social support may not directly address. Acute anxiety may require targeted interventions, including relaxation training, mindfulness‐based therapies, medications, or brief therapist‐delivered interventions (Elsner et al. 2017; Forbes et al. 2023; Halkett et al. 2018).

### 4.1 Limitations

Despite the strengths of the current study, including its longitudinal and prospective design, several limitations should be noted. First, the study relied on patients' subjective perceptions of their distress levels, life satisfaction, and perceived social support. Such self‐reports may be influenced by social desirability bias (Sprangers 1996). However, evidence suggests that subjective perceptions are often more predictive of psychological outcomes than objective measures. For instance, perceived support has been shown to be more beneficial to health outcomes than actual received support (Barrera 2000; Uchino 2004). Thus, although subjective measures are relevant to the study's aims, future research could enhance validity by incorporating non‐self‐report methods (Tashakkori et al. 2020) such as in‐depth interviews, observational approaches, or third‐party reports from radiation oncologists, radiation therapists, nurses, other healthcare professionals (Arnold et al. 2021), or family members.

Second, the sample size for certain cancer subgroups was small, reflecting the limited population in these categories within RT. Most participants were women with breast cancer, which may limit the generalizability of the findings to men or patients with other cancer types, particularly those with low representation in the study.

Third, the study lacked data on non‐responders, and the participation rate was relatively low due to the exclusion criteria and the population characteristics of the radiation center, where many patients are non‐Hebrew speakers. These criteria reduce the generalizability of the findings to non‐Hebrew speakers and other populations.

### 4.2 Future Directions

Future research should consider adopting a positive psychology framework (Casellas‐Grau et al. 2014; Seligman 2002), focusing on well‐being rather than solely on distress. This approach could explore how life satisfaction is influenced by various factors, including social support, particularly among patients who express a desire for PO intervention.

Follow‐up studies might examine distinctions between perceived and received support (Lindorff 2000) and investigate other factors influencing the decision to seek PO intervention, such as pain severity and fatigue, which are commonly experienced by RT patients (Seol et al. 2021). Comparing patients undergoing RT with those not undergoing RT may also help determine whether the observed findings are specific to RT itself.

### 4.3 Theoretical Implications

This study contributes several theoretical insights to the field. First, it shifts the focus from distress as a predictor of the desire for PO intervention to examining whether the desire for PO intervention predicts distress over time. The findings suggest that a simple inquiry at the start of RT can identify patients at greater risk for persistent distress. This finding may have implications for other cancer treatments, such as chemotherapy (Grusdat et al. 2022). However, since this study was conducted on patients undergoing radiotherapy, further research is needed to explore this aspect in future studies.

Second, by employing a moderation model, the study highlights the differential impact of perceived social support on distress and life satisfaction among patients who do and do not desire PO intervention. By examining both distress and positive well‐being outcomes (life satisfaction), the study provides a comprehensive understanding of patient experiences during RT. Notably, the interaction between perceived social support and the desire for PO intervention predicted both reduced depression and increased life satisfaction, underscoring the robustness of these findings across positive and negative outcomes.

### 4.4 Clinical Implications

Screening for psychological distress is a standard practice in oncology (Coppini et al. 2023). However, consistent screening is often hindered by time constraints and limited mental health resources, particularly during the short duration of RT (Adeberg et al. 2021). The findings of this study suggest that a single, straightforward question at the start of RT could serve as an effective tool for identifying at‐risk patients, enabling proactive monitoring and intervention. This approach contrasts with reactive methods that focus on referring patients only after significant distress symptoms emerge (O'Hea et al. 2016).

The PO intervention may be highly relevant for cancer patients across various treatment modalities and throughout the course of the disease. Given that RT often represents the final opportunity for patients to receive psychological support (i.e., RT is often provided as a last oncological intervention or represents the only treatment for patients with cancer) before transitioning to home recovery (Guo et al. 2013), healthcare professionals should prioritize identifying patients with low perceived social support who express a desire for PO intervention. These patients appear to be at greater risk for reduced life satisfaction and increased depression by the end of treatment. Moreover, enhancing the perception of available support from various sources—family, friends, and healthcare professionals—may compensate, to some extent, for the absence of professional PO intervention.

These findings underscore the importance of interpersonal relationships for health and well‐being (Feeney and Collins 2015; Pietromonaco and Collins 2017) and highlight the need to integrate social‐health psychology perspectives (Taylor 2011) into PO practices during RT (Schulze et al. 2022).

## Ethics Statement

The study was approved by the Ethical Committee of the Asota Helsinki Committee, Approval number: AAA‐0099‐22.

## Consent

Participants provided written consent to participate in the study.

## Conflicts of Interest

The authors declare no conflicts of interest.

## Data Availability

The data set is available via link: https://osf.io/9pjnb?view_only=aea44ce5fb19427196fefae7ac53a160.
